# The Protective Role of Curcumin in Osteoarthritis: Establishing Mitochondrial Homeostasis Through Autophagy Induction and Apoptosis Inhibition

**DOI:** 10.3390/ijms27020609

**Published:** 2026-01-07

**Authors:** Kavitha Raja, Rajashree Patnaik, Dineshwary Suresh, Riah Varghese, Adam Eid, Thomas Nau, Yajnavalka Banerjee, Nerissa Naidoo

**Affiliations:** 1 College of Medicine, Mohammed Bin Rashid University of Medicine and Health Sciences, Dubai Health, Dubai P.O. Box 505055, United Arab Emirates; kavitha.raja@dubaihealth.ae (K.R.); rajashree.patnaik@dubaihealth.ae (R.P.); dineshwary.suresh@dubaihealth.ae (D.S.); riah.varghese@dubaihealth.ae (R.V.); 202401049@students.mbru.ac.ae (A.E.); thomas.nau@kch.ae (T.N.); yajnavalka.banerjee@dubaihealth.ae (Y.B.); 2 King’s College Hospital London Dubai, Dubai P.O. Box 28171, United Arab Emirates

**Keywords:** osteoarthritis, chondrocytes, mitochondrial dysfunction, reactive oxygen species, inflammation

## Abstract

Osteoarthritis (OA) is a progressive joint disorder affecting over 250 million people globally and is characterized by chronic pain and disability. Among its key pathogenic mechanisms are mitochondrial dysfunction and elevated reactive oxygen species (ROS), often triggered by inflammatory mediators such as lipopolysaccharide (LPS). This study evaluates the protective effects of curcumin on mitochondrial function, autophagy, and apoptosis in an in vitro model of OA. Human bone marrow-derived mesenchymal stem cells (BMSCs) were differentiated into chondrocytes using MesenCult™-ACF medium. Differentiation was confirmed by histological staining for Type II Collagen, Alcian Blue, and Toluidine Blue. LPS was used to induce an OA-like inflammatory response. Mitochondrial membrane potential (ΔΨm) was assessed using Rhodamine 123 staining. Autophagy and apoptosis were evaluated using Acridine orange and propidium iodide staining, respectively. Western blotting was performed to analyze the expression of pro-caspase-3, Bcl-2, Beclin-1, LC3-I/II, and GAPDH. LPS significantly impaired mitochondrial function, limited autophagy, and enhanced apoptotic signaling (reduced pro-caspase-3). Curcumin (25 µM and 100 µM) restored ΔΨm, increased Beclin-1 and LC3-II, and maintained pro-caspase-3 expression, with Bcl-2 showing a non-monotonic response (higher at 25 µM than at 100 µM). Curcumin exerted cytoprotective effects in inflamed chondrocytes by stabilizing ΔΨm, promoting autophagy, and attenuating apoptotic activation, supporting its multi-target therapeutic potential in OA.

## 1. Introduction

Osteoarthritis is increasingly recognized as a complex, whole-joint disorder involving dynamic and interrelated pathological changes across all articular tissues, including articular cartilage, subchondral bone, synovium, menisci, ligaments, and the infrapatellar fat pad (IFP) [1]. OA is the most prevalent form of arthritis and one of the leading causes of disability worldwide, disproportionately affecting the elderly and physically active populations. Clinically, OA is characterized by chronic pain, joint stiffness, swelling, and reduced mobility, primarily in the hip and knee joints. These symptoms markedly reduce quality of life and functional independence, particularly in aging societies [2].

While degeneration of hyaline cartilage remains a hallmark of OA, the contribution of extra-cartilaginous structures to disease progression is now well-established. The IFP has emerged as a critical yet underappreciated player in OA pathogenesis. It undergoes inflammation, fibrosis, and vascular remodeling, leading to increased secretion of pro-inflammatory adipokines and cytokines such as IL-6, leptin, and MCP-1, which exacerbate synovitis and cartilage degradation [3]. Moreover, IFP inflammation is associated with both peripheral and central sensitization, thereby contributing to OA-related pain and disability [1].

In parallel, biomechanical stress plays a crucial role in OA onset and progression. Altered joint loading due to malalignment, obesity, or trauma induces mechano-transduction signals in chondrocytes that activate catabolic pathways. This results in matrix breakdown, cytoskeletal remodelling, mitochondrial dysfunction, and abnormal autophagic responses [4]. Changes in cartilage stiffness and deformation also compromise load distribution and further promote chondrocyte hypertrophy and apoptosis. Collectively, these biochemical and biomechanical interactions among multiple joint compartments create a self-perpetuating cycle of tissue degeneration and inflammation in knee OA [5].

Structurally, OA affects weight-bearing joints in which opposing bones are covered in articular cartilage and enclosed in a capsule lined by synovium [6]. The disease is marked histopathological by progressive loss of articular cartilage, subchondral bone sclerosis, osteophyte formation, synovial inflammation, and fibrosis, all of which culminate in joint space narrowing and functional impairment [7]. The severity and progression of these pathological changes are influenced by age, gender (notably female predominance), obesity, excessive physical activity, trauma, and genetic predisposition [8].

Several recent studies have explored the therapeutic effects of curcumin in OA models, highlighting its multi-targeted role in modulating key cellular pathways. Curcumin was reported to promote autophagy, suppress apoptosis, and preserve mitochondrial function in chondrocytes exposed to pro-inflammatory stimuli such as IL-1β and LPS [9]. It also attenuated oxidative stress, inhibited the NF-κB and MAPK signaling pathways, and restored mitochondrial membrane potential (ΔΨm), thereby improving chondrocyte survival and reducing cartilage degradation [10]. Despite these advancements, most studies focused on individual signaling axes or used limited endpoint analyses. Therefore, the present study aimed to provide a more integrative assessment of curcumin’s cytoprotective potential by evaluating mitochondrial homeostasis, autophagic flux, and apoptotic signaling in a chondrocyte inflammation model using both fluorescence imaging and Western blot analysis [11].

Despite extensive research, the pathogenesis of OA remains incompletely understood. Chondrocytes, the sole resident cells of articular cartilage, play a pivotal role in maintaining the extracellular matrix (ECM). However, due to their avascular and aneural environment and inherently limited regenerative capacity, these cells are vulnerable to metabolic stress and mechanical overload. During OA progression, chondrocytes undergo phenotypic changes resembling hypertrophic differentiation. This results in the secretion of catabolic enzymes (e.g., MMPs and ADAMTS), pro-inflammatory cytokines (e.g., IL-1β, TNF-α), and nitric oxide, which further degrade ECM components and amplify the inflammatory cascade [12].

Recently, mitochondrial dysfunction has emerged as a critical contributor to the molecular pathology of OA. Chondrocytes from OA cartilage display altered mitochondrial morphology, reduced oxidative phosphorylation capacity, decreased ATP production, and elevated generation of mitochondrial reactive oxygen species (ROS) [10]. The diminished ΔΨm in OA chondrocytes is not only a hallmark of early dysfunction but also a therapeutic target for mitochondrial protection [13].

Advances in fluorescence imaging and high-resolution microscopy have enabled real-time visualization of mitochondrial dynamics, allowing researchers to investigate changes in ΔΨm and autophagic flux in response to cellular stress in osteoarthritic chondrocytes [14]. Through these techniques, mitochondrial impairment has been linked to defective mitophagy, impaired energy metabolism, and chondrocyte apoptosis, making mitochondrial homeostasis a focal point in OA pathogenesis [15].

Therefore, the present study aimed to evaluate the alterations in mitochondrial membrane potential in LPS-induced osteoarthritic chondrocytes and to investigate the potential protective role of curcumin, a naturally derived polyphenol with known anti-inflammatory and antioxidant properties, in restoring mitochondrial function [16]. This study investigated the cytoprotective effects of curcumin on inflamed chondrocytes, with a focus on its ability to modulate mitochondrial function, autophagic flux, and apoptotic signaling pathways under inflammatory stress.

## 2. Results

### 2.1. Fluorescence Imaging and Chondrocyte Differentiation in an In Vitro Osteoarthritic Model

**Figure 1:**
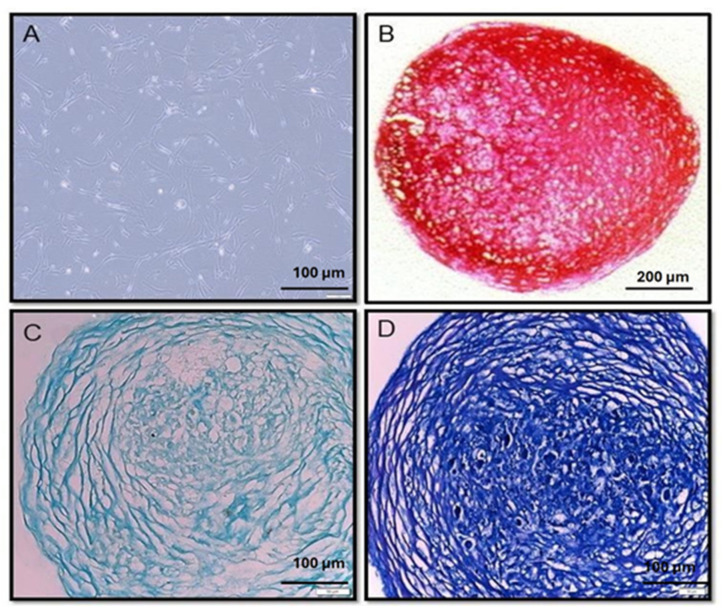
Differentiation of Chondrocytes from Human Bone Marrow Mesenchymal Stem Cells (BMSC) after 28 Days in Culture. (**A**). Control chondrocytes. (**B**). Chondrocytes after type II collagen staining. (**C**). Chondrocytes after alcian blue staining. (**D**). Chondrocytes after toluidine blue staining.

As shown in Figure 1, chondrocytes were successfully differentiated from BMSCs using chondrogenic differentiation media, and their identity was confirmed through histological staining with Alcian Blue and Toluidine Blue. Alcian Blue revealed the presence of proteoglycans, including Aggrecan, and collagens Type II and X, indicating effective differentiation after 21 d. Toluidine Blue staining further confirmed the presence of glycosaminoglycans (GAGs) in the extracellular matrix (ECM) by exhibiting metachromasia a shift from blue to purple in areas rich in GAGs providing a visual indication of their distribution and density. This metachromatic change served as a qualitative marker of ECM maturation and chondrocyte phenotype.

Following differentiation, a pro-inflammatory model was established by exposing the chondrocytes to LPS. Alcian Blue, a cationic dye that binds specifically to negatively charged sulfate groups on GAGs such as chondroitin sulfate and keratan sulfate, was again used to assess ECM composition. These GAGs are key structural components of cartilage, contributing to its gel-like consistency and ability to resist compressive forces.

Chondrogenic differentiation was confirmed by the expression of key chondrocyte markers, including proteoglycans (Aggrecan) and collagen types II and X. Specifically, Type II collagen was detected through immunohistochemical staining, as detailed in the methodology section and illustrated in Figure 1.

### 2.2. Effect of Curcumin on Mitochondrial Membrane Potential (ΔΨm) in LPS-Challenged Chondrocytes

**Figure 2:**
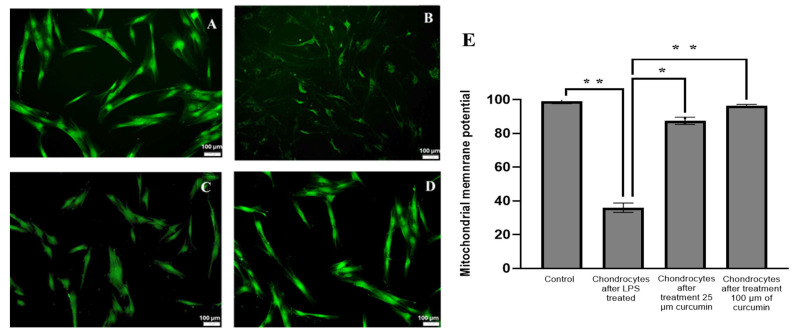
Effect of Curcumin on ΔΨM in a Pro-Inflammatory Chondrocyte Model as Demonstrated by Rhodamine 123 Staining and Visualization via Fluorescence Microscopy (**A**). Control chondrocytes. (**B**). Chondrocytes after 10 µg/mL Lipopolysaccharide (LPS) treatment. (**C**). Chondrocytes treated with 100 µM Curcumin in the presence of LPS. (**D**). Quantitative analysis of ΔΨm expressed as percentage fluorescence intensity relative to control, measured using ImageJ software. Data are presented as mean ± SD from three independent experiments. (**E**). Quantitative histogram showing relative fluorescence intensity. Data were presented as mean ± SD from *n* = 3 independent biological replicates, with fluorescence intensity quantified in triplicate within each replicate Statistical analysis was performed using one-way ANOVA followed by Tukey’s post hoc test. Asterisks indicate levels of statistical significance: *p* < 0.05 (*), *p* < 0.01 (**) compared to the LPS-treated group.

LPS-treated chondrocytes showed a marked reduction in ΔΨm as indicated by decreased green fluorescence. Curcumin treatment restored mitochondrial function, with the 100 μM concentration producing a more pronounced recovery compared to the LPS group. As illustrated in Figure 2, exposure to LPS resulted in a significant reduction in ΔΨm when compared to LPS-naïve cells. This reduction was statistically significant (*p* < 0.05) compared to both the LPS-only and curcumin-treated groups. However, treatment with curcumin led to a notable increase in mitochondrial membrane potential, suggesting that curcumin helps counteract the detrimental effects of LPS on chondrocytes (Figure 2).

Fluorescent images were captured using an Olympus fluorescence microscope (Olympus, Tokyo, Japan), providing high-resolution visualizations of the stained cells. These images were analyzed to quantify changes in ΔΨm by measuring the fluorescence intensity of Rhodamine 123. ImageJ software was utilized for this quantitative analysis, allowing for precise measurement of the fluorescence emitted by the stained mitochondria.

The dissipation of ΔΨm, indicative of mitochondrial dysfunction, was represented as a percentage reduction in green fluorescence intensity of Rhodamine 123. This decrease in fluorescence intensity is directly correlated with the loss of ΔΨm. By comparing the fluorescence intensities under the LPS-only condition and the LPS-plus curcumin condition, the protective effect of curcumin against LPS-induced ΔΨm disruption was assessed. This analysis demonstrated that curcumin played a significant role in preserving mitochondrial function under inflammatory stress, as evidenced by the maintained fluorescence intensity in treated cells [17].

The increase in ΔΨm following curcumin treatment indicates its potential protective role against LPS-induced mitochondrial dysfunction and cellular stress in chondrocytes. This protective effect is crucial as it implies that curcumin can maintain mitochondrial integrity and function under inflammatory conditions induced by LPS. The data supports the hypothesis that curcumin can mitigate MMP (ΔΨm) dissipation, thereby enhancing cell survival and function [18].

In a comparative evaluation of the efficacy of the different treatments, curcumin emerged as the most potent modulator of ΔΨm. The superior efficacy of curcumin highlights its therapeutic potential in protecting chondrocytes from LPS-induced mitochondrial dysfunction and apoptosis. This finding underscores the promising role of curcumin in managing inflammatory responses and preserving mitochondrial health in chondrocytes subjected to pro-inflammatory stimuli [19].

### 2.3. Detection of Autophagy by Acridine Orange Staining in Curcumin-Treated Chondrocytes

**Figure 3:**
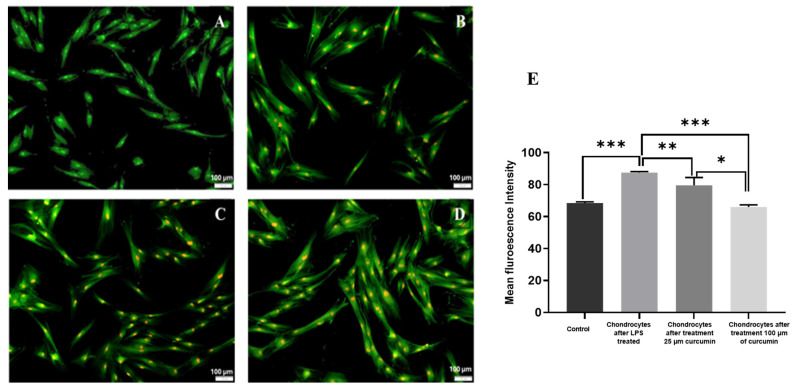
Detection of Autophagy by acridine Orange staining acidic vesicular organelles with the treatment of curcumin, (**A**). Control chondrocytes. (**B**). Chondrocytes after LPS treated. (**C**). Chondrocytes after treatment 25 µm curcumin. (**D**). Chondrocytes after treatment 100 µm of curcumin. (**E**). Quantitative histogram representing the percentage of AO-positive acidic vesicular organelles (AVOs), determined from fluorescence intensity analysis in ImageJ. Data were presented as mean ± SD from *n* = 3 independent biological replicates, with fluorescence quantification performed in triplicate within each replicate. Statistical significance was determined using one-way ANOVA followed by Tukey’s post hoc test; *p* < 0.05 was considered significant. Asterisks indicate levels of statistical significance: *p* < 0.05 (*), *p* < 0.01 (**), *p* < 0.001 (***) compared to the LPS-treated group.

To assess the autophagic activity in chondrocytes under inflammatory stress, Acridine Orange (AO) staining was employed to visualize the formation of acidic vesicular organelles (AVOs) [20]. As shown in Figure 3, exposure to LPS resulted in a marked decrease in red-orange fluorescence intensity, indicative of diminished autophagic activity in the chondrocytes. This reduction in AO-positive staining reflects the suppression of autophagosome and autolysosome formation under inflammatory conditions. An increased red fluorescence signal in curcumin-treated groups, relative to the LPS treated group, indicated enhanced formation of AVOs, suggesting that curcumin induced autophagy restoration under inflammatory conditions.

Following treatment with 100 µM curcumin for 24 h, a significant enhancement in red fluorescence was observed, suggesting the restoration of autophagic flux. The curcumin-treated group exhibited numerous red-stained AVOs dispersed throughout the cytoplasm, indicating increased acidification and autophagic vesicle formation (Figure 3).

This result highlights curcumin’s capacity to counteract LPS-associated reduction in autophagy in chondrocytes. The increase in AO-positive vesicles is consistent with previous findings where curcumin has been shown to induce autophagy by modulating intracellular pH and lysosomal biogenesis [21]. The elevation in autophagic activity in curcumin-treated cells suggests its potential to alleviate cellular stress and maintain homeostasis through enhanced degradation of damaged organelles and proteins.

These results confirmed that curcumin exerted a potent stimulatory effect on autophagy in LPS-treated chondrocytes, positioning it as a promising agent in the modulation of autophagy-related pathways in OA.

### 2.4. Evaluation of Chondrocyte Viability by Propidium Iodide Staining Following Curcumin Treatment

To evaluate the cytoprotective effect of curcumin against lipopolysaccharide (LPS)-induced cytotoxicity, chondrocyte viability was assessed using propidium iodide (PI) staining. PI is a membrane-impermeable dye that binds to DNA in membrane-compromised cells, emitting bright red fluorescence in apoptotic or necrotic cells [9].

**Figure 4:**
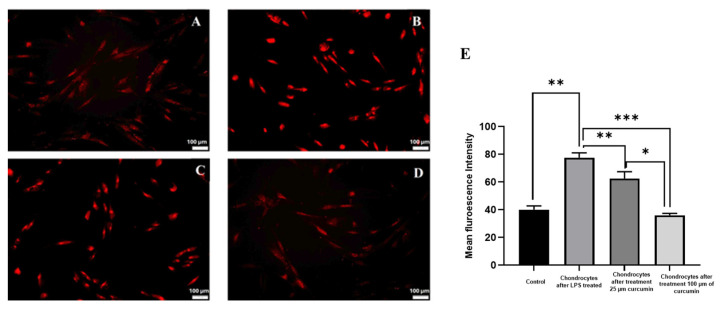
Observation of chondrocyte viability via propidium iodide staining with the treatment of curcumin, (**A**). Control chondrocytes. (**B**). Chondrocytes after LPS treated. (**C**). Chondrocytes after treatment 25 µm curcumin. (**D**). Chondrocytes after treatment 100 µM of curcumin. (**E**). Quantitative histogram representing the percentage of PI-positive cells from fluorescence intensity analysis. Data were presented as mean ± SD from *n* = 3 independent biological replicates, with fluorescence quantification performed in triplicate within each replicate. Statistical significance was determined using one-way ANOVA followed by Tukey’s post hoc test; *p* < 0.05 (*), *p* < 0.01 (**), *p* < 0.001 (***) vs. LPS-treated group.

As shown in Figure 4, LPS treatment led to a marked increase in the number of PI-positive nuclei, indicating significant membrane damage and cell death. This result confirmed the cytotoxic nature of LPS in mimicking an inflammatory osteoarthritic environment. In contrast, curcumin-treated chondrocytes exhibited a substantial reduction in red fluorescence compared to the LPS treated group, reflecting enhanced membrane integrity and improved cell survival (Figure 4).

The fluorescence intensity reflects the proportion of cells exhibiting loss of plasma membrane integrity, which is indicative of reduced cell viability under inflammatory stress. Notably, both 25 µM and 100 µM curcumin reduced the proportion of PI-positive cells relative to the LPS-treated group. The 100 µM concentration maintained this protective response, indicating preservation of cell membrane integrity under inflammatory stress. These findings suggest that curcumin helps maintain chondrocyte viability by reducing LPS-induced membrane damage and cellular stress. These findings proved that curcumin may mitigate LPS-induced chondrocyte apoptosis and protect cellular viability under oxidative stress. Compared to the LPS treated group, curcumin treated cells demonstrated a notable reduction in PI positive staining, suggesting an improvement in chondrocyte membrane integrity and viability. It also indicated that curcumin contributes to maintaining chondrocyte viability by counteracting inflammation-mediated membrane damage, reinforcing its therapeutic potential in the management of osteoarthritic degeneration.

### 2.5. Western Blot Analysis of Apoptotic and Autophagy Markers in Curcumin-Treated Chondrocytes

To investigate the molecular mechanisms underlying curcumin’s protective role in inflammatory chondrocytes, Western blot analysis was conducted to evaluate the expression of apoptosis and autophagy related proteins, including Bcl-2, Pro-caspase-3, Beclin-1, and LC3-I/II.

**Figure 5:**
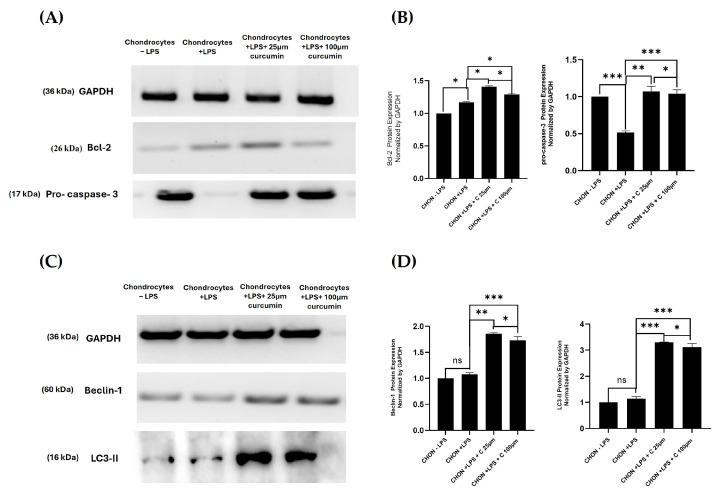
Curcumin modulates apoptotic and autophagic protein expression in LPS-stimulated chondrocytes. (**A**) Representative Western blot images showing the expression of pro-caspase-3 and Bcl-2 in control (–LPS), LPS-treated, and curcumin-treated (25 µM and 100 µM) chondrocytes. Curcumin restored the pro-caspase-3 band intensity that was reduced by LPS, indicating inhibition of caspase cleavage and attenuation of apoptosis. It also upregulated the anti-apoptotic protein Bcl-2 at 25 µM, whereas a downregulation was observed at 100 µM, suggesting feedback modulation between apoptotic and autophagic signaling. (**B**) Densitometric analysis of pro-caspase-3 and Bcl-2 expression normalized to GAPDH (*n* = 3). Data were presented as mean ± SD from *n* = 3 independent biological replicates, with band intensity quantification performed in triplicate within each replicate. Statistical significance was determined using one-way ANOVA followed by Tukey’s post hoc test; * *p* < 0.05, ** *p* < 0.01, *** *p* < 0.001, ns = not significant. (**C**) Representative Western blot images showing the expression of Beclin-1 and LC3-II in control (–LPS), LPS-treated, and curcumin-treated (25 µM and 100 µM) chondrocytes. LPS exposure did not significantly alter the Beclin-1 or LC3-II expression, indicating that basal autophagic activity was largely maintained under inflammatory conditions. Curcumin treatment upregulated Beclin-1 and LC3-II levels at 25 µM but showed a moderate decrease at 100 µM, consistent with the expression pattern of Bcl-2, suggesting a coordinated regulation between autophagy and apoptosis pathways. (**D**) Densitometric analysis of Beclin-1 and LC3-II normalized to GAPDH. Data were presented as mean ± SD from *n* = 3 independent biological replicates, with quantification performed in triplicate for each replicate. Statistical significance was evaluated using one-way ANOVA followed by Tukey’s post hoc test; * *p* < 0.05, ** *p* < 0.01, *** *p* < 0.001, ns = not significant.

As shown in Figure 5, exposure to lipopolysaccharide (LPS) led to a decrease in pro-caspase-3 band intensity, indicating cleavage of the inactive precursor into its active form and activation of apoptosis. In contrast, treatment with curcumin at 25 µM and 100 µM restored the pro-caspase-3 band intensity, suggesting that curcumin inhibited caspase-3 cleavage and thereby attenuated apoptotic activation.

Curcumin also modulated Bcl-2 expression, a protein with a dual role in both apoptosis inhibition and autophagy regulation. A pronounced increase in Bcl-2 was detected at 25 µM, consistent with strong anti-apoptotic and cytoprotective activity, whereas a slight decrease at 100 µM suggested a feedback balance between apoptotic suppression and autophagic activation.

With respect to autophagy markers, Beclin-1 and LC3-II levels did not differ significantly between the control and LPS treated groups, indicating that basal autophagy was largely preserved under inflammatory stress. However, curcumin treatment significantly enhanced Beclin-1 and LC3-II expression, confirming activation of autophagic flux in a concentration-responsive manner. Collectively, these results demonstrated that curcumin mitigated LPS-induced apoptotic activation by maintaining pro-caspase-3 expression and promoting autophagy, thereby supporting a coordinated cytoprotective mechanism that helps preserve chondrocyte homeostasis.

## 3. Discussion

This study aimed to assess the effects of curcumin on ΔΨm in chondrocytes derived from BMSCs under pro-inflammatory conditions. The successful differentiation of BMSCs into chondrocytes was confirmed through histological staining with Collagen Type II, Alcian Blue, and Toluidine Blue. These stains demonstrated the presence of key cartilage components such as proteoglycans, collagen types II and X, and GAGs, validating the chondrocyte phenotype and the effectiveness of the differentiation process.

Upon inducing inflammation by exposing differentiated chondrocytes to LPS, we observed a significant reduction in ΔΨm, confirming mitochondrial dysfunction, which is a hallmark of inflammation-associated cartilage degradation in OA.

LPS-induced stress is known to impair mitochondrial function, triggering a cascade of events leading to cellular apoptosis and further exacerbating cartilage degradation in OA. In this context, the observation of reduced ΔΨm in LPS-challenged chondrocytes highlights the vulnerability of mitochondria to inflammatory insults [13].

Treatment with curcumin, a polyphenolic compound known for its anti-inflammatory and antioxidant properties, significantly restored ΔΨm in LPS-exposed chondrocytes. This result suggests that curcumin acts as a potent mitochondrial protective agent, maintaining mitochondrial integrity and function under inflammatory stress. The increase in ΔΨm following curcumin treatment further supports the hypothesis that curcumin can mitigate mitochondrial dysfunction and prevent apoptosis in chondrocytes subjected to inflammatory stimuli [22].

Comparative analysis showed that curcumin was the most effective modulator of ΔΨm among the treatments tested, emphasizing its potential therapeutic role in OA. Curcumin’s ability to preserve mitochondrial function in the face of inflammatory stress underscores its value in managing the mitochondrial dysfunction associated with OA pathogenesis. Moreover, this study suggests that curcumin may provide a promising avenue for therapeutic intervention aimed at reducing mitochondrial damage, oxidative stress, and apoptosis in chondrocytes, ultimately mitigating cartilage degradation and improving clinical outcomes for OA patients.

Upon LPS induced inflammation, we observed a marked decrease in ΔΨm, indicative of mitochondrial dysfunction, a key pathological feature in OA [19]. Treatment with curcumin (100 μM) successfully restored ΔΨm, suggesting its role as a potent mitochondrial stabilizer and anti-apoptotic agent. These findings support earlier reports of curcumin’s ability to preserve mitochondrial function under oxidative and inflammatory stress [23]. In a hierarchical evaluation of efficacy, curcumin emerged as the most potent modulator among the tested bioactive compounds, underscoring its significant potential in mitigating the adverse effects of LPS on chondrocytes [24].

Curcumin restored autophagic activity in LPS-treated chondrocytes, as evidenced by increased AVO formation and enhanced red-orange fluorescence in AO staining. Cell viability and membrane integrity were further investigated via PI staining. LPS exposure resulted in a substantial increase in red nuclear fluorescence, indicative of membrane-compromised, non-viable cells. Following curcumin treatment, a significant reduction in PI-positive cells was observed, implying that curcumin attenuated LPS-induced necrotic and apoptotic death. These results confirm that curcumin’s have anti-apoptotic properties and have capacity to scavenge ROS and stabilize mitochondrial membranes [25].

Moreover, to elucidate the molecular pathways involved, Western blot analysis was performed to assess the expression of apoptosis-related (Bcl-2, pro-caspase-3) and autophagy-related (Beclin-1, LC3-II) proteins. LPS treatment markedly decreased pro-caspase-3 expression, indicating its cleavage into the active form and subsequent activation of apoptosis, while Bcl-2 expression was elevated, reflecting an imbalance in apoptotic regulation under inflammatory stress. In parallel, the autophagic markers Beclin-1 and LC3-II were reduced, suggesting a suppression of autophagic flux.

Curcumin treatment effectively reversed these molecular alterations, displaying differential modulation of apoptosis- and autophagy-related proteins. In LPS-treated chondrocytes, the reduction in pro-caspase-3 indicated cleavage into its active form, signifying caspase-3 activation and apoptotic induction. Upon curcumin treatment, restoration of pro-caspase-3 expression suggested inhibition of this LPS-induced caspase activation and attenuation of apoptosis. Bcl-2 protein expression exhibited a non-linear trend strongly increased at 25 µM and slightly reduced at 100 µM indicating a regulatory balance between cytoprotective and autophagic responses. Concurrently, curcumin markedly enhanced Beclin-1 and LC3-II expression, confirming activation of autophagic signaling. These molecular changes highlight curcumin’s dual modulatory effect anti-apoptotic through suppression of LPS-induced caspase activation and pro-autophagic through restoration of autophagy signaling in osteoarthritic chondrocytes [26].

The apparent increase in both Bcl-2 (anti-apoptotic) and Beclin-1 (pro-autophagic) following curcumin treatment underscores Bcl-2’s dual regulatory role, as it interacts with Beclin-1 to coordinate the balance between apoptosis inhibition and autophagy induction. At lower concentrations (25 µM), curcumin strongly enhanced Bcl-2 expression, promoting cytoprotection, while at higher concentrations (100 µM), a slight decline in Bcl-2 coincided with a proportional increase in autophagic markers, suggesting feedback regulation between these pathways.

These findings are consistent with previous reports suggesting that curcumin exerts anti- apoptotic effects by modulating Bcl-2 family proteins and autophagy-related mediators in chondrocytes and other cell types [27]. The upregulation of autophagy markers such as LC3-II and Beclin-1, along with the down regulation of apoptotic indicators, supports the hypothesis that curcumin confers cytoprotective effects by modulating the interplay between pro-survival signaling and apoptotic pathways, thereby maintaining cellular homeostasis under inflammatory conditions. Thus, the results of the Western blot provided molecular evidence that curcumin ameliorates LPS-induced cellular stress by promoting autophagy and attenuating apoptosis in osteoarthritic chondrocytes. Integrating fluorescence microscopy with flow cytometry or mass spectrometry could yield more precise data. Additionally, super resolution microscopy, in vivo imaging, and machine learning can enhance monitoring, analysis, and clinical relevance.

Nevertheless, this study indicated an interplay between mitochondrial dysfunction, inflammation, and OA progression. Understanding these processes and their underlying mechanisms is crucial for developing targeted therapeutic interventions aimed at preserving joint health and alleviating OA’s burden. Further research is warranted to elucidate the complex signaling pathways involved and identify novel therapeutic targets for OA management.

This study offers valuable insights into the anti-inflammatory and cytoprotective effects of curcumin in an in vitro model of OA, particularly its ability to preserve ΔΨm, enhance autophagy, and inhibit apoptosis in LPS-induced chondrocyte inflammation. The use of multiple complementary assays including fluorescence microscopy, Western blotting, and viability staining strengthens the reliability of the findings. However, the study is limited by its in vitro design, which may not fully replicate the complex in vivo joint environment. Additionally, curcumin’s poor bioavailability in physiological conditions was not addressed, which could affect translational relevance. Future investigations should include in vivo studies and pharmacokinetic assessments to better understand the clinical applicability of curcumin in OA management.

## 4. Materials and Methods

### 4.1. Study Design and Landscape

This in vitro investigation utilized a cross-sectional design to examine the immediate influence of curcumin on inflammatory processes in human chondrocytes. Endpoint assessments included ΔΨm, autophagic activity, and apoptotic cell death. These were evaluated using Rhodamine 123, Acridine Orange, and Propidium Iodide staining, respectively, supported by fluorescence microscopy. Additionally, Western blot analysis quantified the expression of key regulatory proteins Bcl-2, Caspase-3, Beclin-1, and LC3-I/II to mechanistically validate the cellular responses. This approach provided a targeted overview of curcumin’s anti-inflammatory and cytoprotective potential in OA without requiring extended temporal assessment.

### 4.2. Differentiation of Chondrocytes from BMSCs

Human bone marrow mesenchymal stem cells (BMSCs) were obtained from AddexBio Technologies (San Diego, CA, USA) and shipped in liquid nitrogen. Upon arrival, the cells were thawed and washed with phosphate-buffered saline (PBS; Gibco, Thermo Fisher Scientific, Waltham, MA, USA) to remove the cryoprotectant dimethyl sulfoxide (DMSO). The BMSCs were then cultured in mesenchymal stem cell growth medium (MSCM, AddexBio, CA, USA), supplemented with 10% fetal bovine serum (FBS; HiMedia Laboratories, Mumbai, India) and 1% penicillin-streptomycin (HiMedia Laboratories, Mumbai, India). Cultures were maintained at 37 °C in a 5% CO_2_ incubator, and the medium was replaced every three days. Cell growth and confluency were monitored regularly. Once the cells reached 90% confluency, they were passaged using trypsin-EDTA (HiMedia Laboratories, Mumbai, India) and expanded in fresh MSCM.

The chondrogenic differentiation of BMSCs was performed according to the pellet culture method described by Patnaik et al. [28], which involved the use of MesenCult™-ACF medium (STEMCELL Technologies Inc., Vancouver, BC, Canada) for 21 d with regular medium changes to promote chondrogenesis. BMSCs that underwent five passages (Passage 5) were harvested to begin the differentiation process. Using an automated cell counter (CellDrop™, DeNovix Inc., Wilmington, DE, USA), a cell count of 2 × 10^6^ BMSCs was obtained. These cells were then pelleted by centrifugation at 1000 rpm for 10 min in 15 mL polypropylene tubes. About 2 mL of complete MesenCult™-ACF chondrogenic differentiation medium was added to each cell pellet. Subsequently, 0.5 mL of this suspension was transferred into four separate 15 mL polypropylene tubes. These tubes were again centrifuged at 1000 rpm for 10 min at 25 °C using a Thermoscientific, USA 400R centrifuge (Thermo Fisher Scientific, Waltham, MA, USA). The cell pellets were then incubated at 37 °C in a 5% CO_2_ environment for three days. After the initial incubation period, the medium was replaced, and the cells were returned to the same incubation conditions for an additional 21 d. During this time, the medium was changed every three days to ensure optimal differentiation conditions. When refreshing the medium, the cell pellets were gently flicked to prevent them from sticking to the surfaces of the tubes.

### 4.3. Histological Sample Preparation

The histological process began by fixing the cell pellets in 10% neutral-buffered formalin for 24 h to preserve both cellular and extracellular components. After fixation, the samples were dehydrated through a graded ethanol series to remove water. This was followed by clarification with xylene to replace the ethanol and make the tissue transparent. The samples were then embedded in paraffin to provide structural support for thin sectioning.

Paraffin blocks were sectioned at a thickness of 6 μm using a Leica RM2255 rotary microtome (Leica Biosystems, Nussloch, Germany), which allowed optimal visualization of cellular details. The sections were stained using Alcian Blue and Toluidine Blue, following established protocols with minor modifications [29]. Alcian Blue highlighted acidic polysaccharides like glycosaminoglycans, while Toluidine Blue stained acidic tissue elements, enhancing the visibility of cellular architecture and the extracellular matrix (ECM) [30].

To detect Type II collagen in the ECM, rehydrated sections were incubated overnight at 4 °C with an anti-collagen II antibody (rabbit, Antibodies Online GmbH, Aachen, Germany) diluted in 2% bovine serum albumin (BSA; Sigma-Aldrich, St. Louis, MO, USA). After washing with PBS to remove unbound antibody, the sections were incubated at room temperature for 1 h with a horseradish peroxidase-conjugated secondary antibody (Abcam, Cambridge, UK). Following another PBS wash, the sections were counterstained with hematoxylin (Merck KGaA, Darmstadt, Germany) and examined under a light microscope to confirm the presence of Type II collagen [31].

### 4.4. Microscopic Analysis

Chondrocyte spheroids were fixed in 4% paraformaldehyde for 15 min and washed with phosphate-buffered saline (PBS). They were then embedded in paraffin, sectioned at 5 μm thickness, and mounted onto glass slides. Sections were deparaffinized, rehydrated, and stained with Hematoxylin and Eosin (H&E) to evaluate cellular morphology. To assess matrix production, additional sections were stained with Alcian Blue and Toluidine Blue. Microscopic images were captured using a light microscope (Olympus BX51, Tokyo, Japan) equipped with a digital camera [32].

### 4.5. Establishing a Pro-Inflammatory Model

To develop a pro-inflammatory model, chondrocytes were treated with 10 µg/mL LPS for 24 h [28]. The LPS dose of 10 µg/mL was selected based on MTT assay results, ensuring effective induction of inflammatory pathways without triggering hyperinflammation or compromising cell viability

### 4.6. Evaluation of Mitochondrial Membrane Potential (ΔΨm)

Rhodamine 123 (Rh123; Cat# R8004, Sigma-Aldrich, St. Louis, MO, USA), a positively charged fluorescent dye, was employed for the targeted labeling of active mitochondria. This dye is utilized for real-time monitoring of mitochondrial activity within live cells as the distribution of it aligns with the negative charge gradient across the inner mitochondrial membrane. Loss of ΔΨm is a hallmark of mitochondrial dysfunction and is typically detected by decreased Rhodamine 123 fluorescence intensity [33]. Decrease in the ΔΨm leads to dye loss and subsequently diminished fluorescence. Mitochondrial energization also induces quenching of RH-123 fluorescence, and the rate of fluorescence decay is proportional to the ΔΨm [34].

The ΔΨm created by proton pumps in Complexes I, III, and IV plays a crucial role in energy storage during oxidative phosphorylation. To assess how different treatments impact the ΔΨm, a staining protocol using Rhodamine 123 was employed in this study. Rhodamine 123 is particularly effective for various fluorescence-based applications due to its distinct excitation and emission wavelengths. Specifically, Rhodamine 123 has a peak excitation wavelength of 508 nm and a peak emission wavelength of 528 nm [35].

In an endeavor to elucidate the potential modulatory effects of curcumin on ΔΨm, chondrocytes were exposed to LPS. “Curcumin (Sigma-Aldrich, St. Louis, MO, USA) was first dissolved in dimethyl sulfoxide (DMSO) to prepare a 100 mM stock solution, which was then diluted in culture medium to the desired working concentrations (25 µM and 100 µM). The final DMSO concentration did not exceed 0.1% (*v*/*v*) in any experimental condition, including controls.” These cells were subsequently treated with curcumin (100 µM) over a 24 h period, followed by an assessment using the Rhodamine 123 staining assay. LPS-induced diminution in ΔΨm was observed versus LPS-naive cells. However, the concomitant administration of curcumin led to a discernible augmentation in this potential. This suggested that curcumin may exert a protective role against LPS-induced chondrocyte apoptosis [36].

To evaluate the impact of curcumin on ΔΨm, a Rhodamine 123 staining assay was performed. Chondrocytes were cultured in 24-well plates and treated with 100 µM curcumin in the presence of 10 µg/mL lipopolysaccharide (LPS). After the treatment period, cells were washed thoroughly with phosphate-buffered saline (PBS) to remove residual media and treatment compounds. They were then incubated with 2 μM Rhodamine 123 (Thermo Fisher, Waltham, MA, USA) for 15 min at room temperature in the dark. Following staining, excess dye was removed with PBS washes, and fluorescence intensity was visualized using a fluorescence microscope Olympus fluorescence microscope (Olympus, Tokyo, Japan) to assess ΔΨm [37]. All ΔΨm measurements were obtained from *n* = 3 independent biological replicates, and each biological replicate was analyzed in technical triplicate to ensure reproducibility.

### 4.7. Detection of Autophagy by Acridine Orange Staining

To assess autophagy induction in chondrocytes, acidic vesicular organelles (AVOs) were visualized using Acridine Orange (AO) staining. Acridine Orange is a lysosomotropic metachromatic fluorophore that differentially stains cytoplasm and acidic organelles based on pH. In neutral pH environments, AO emits green fluorescence when bound to nucleic acids; however, upon accumulation in acidic vesicles such as autophagosomes and autolysosomes, it emits bright red/orange fluorescence due to protonation and subsequent aggregation [38].

In this staining, the BMSCs were differentiated into chondrocytes, and inflammation was induced using 10 μg/mL of LPS. Cells were subsequently treated with 100 μM curcumin for 24 h to examine its modulatory effect on autophagy.

Following treatment, cells were cultured in 24-well plates, washed thrice with PBS, and incubated with 1 μg/mL Acridine Orange (Sigma-Aldrich, St. Louis, MO, USA) for 15 min at 37 °C in the dark. After staining, excess dye was removed by rinsing the cells three times with PBS. High-resolution images were obtained using an Olympus fluorescence microscope (Olympus, Tokyo, Japan) [39].

The red-to-green fluorescence ratio was used as a semi-quantitative marker of autophagic vesicle formation. ImageJ software version 1.53t (NIH, Bethesda, MD, USA) was employed to quantify fluorescence intensity across all experimental groups, allowing for comparative analysis of autophagy levels. Acridine Orange staining experiments were performed using *n* = 3 independent biological replicates, with technical triplicate imaging fields analyzed for each replicate

### 4.8. Observation of Chondrocyte Viability via Propidium Iodide Staining

To evaluate membrane integrity and assess cell viability, propidium iodide (PI) staining was employed. PI is a fluorescent intercalating agent that is impermeable to live cells and selectively binds to DNA in cells with compromised plasma membranes, thereby serving as a reliable marker for necrosis or late-stage apoptosis [40].

In this staining, differentiated chondrocytes derived from BMSCs were seeded into 24-well plates and exposed to 10 µg/mL of LPS to induce an inflammatory state resembling osteoarthritic conditions. To investigate the potential cytoprotective role of curcumin, the LPS-stimulated chondrocytes were treated with 100 µM Curcumin for 24 h [41].

Following treatment, cells were washed thrice with PBS to eliminate residual compounds. Subsequently, the cells were incubated with 10 μg/mL PI (Sigma-Aldrich, St. Louis, MO, USA) in PBS for 15 min in the dark at room temperature. Excess dye was removed by additional PBS washes to minimize background fluorescence [42].

Fluorescence microscopy was performed using an Olympus fluorescence microscope (Olympus, Tokyo, Japan) to capture images of stained cells. PI-positive nuclei, indicative of membrane compromised cells, emitted bright red fluorescence. Quantitative analysis of the fluorescence intensity was conducted using ImageJ software. PI staining assays were conducted using *n* = 3 biological replicates, and fluorescence quantification was performed across three technical fields per replicate.

### 4.9. Western Blot Analysis of Apoptotic and Autophagy Markers in Chondrocytes

To evaluate the modulatory effects of curcumin on apoptosis and autophagy signaling pathways, Western blot analysis was performed to assess the expression of key regulatory proteins. These included B-cell lymphoma 2 (Bcl-2), Pro-caspase-3, Beclin-1, and microtubule-associated protein light chain 3 (LC3-I/II), with glyceraldehyde 3-phosphate dehydrogenase (GAPDH) used as the internal loading control [43].

Differentiated chondrocytes were treated with 10 μg/mL LPS to induce inflammation and co-treated with 25 μM or 100 μM curcumin for 24 h. Following treatment, cells were lysed using RIPA buffer (Thermo Fisher Scientific, Waltham, MA, USA) supplemented with protease and phosphatase inhibitors. The protein content of each lysate was quantified using the bicinchoninic acid (BCA) assay [44].

Equal amounts of protein (20 μg) from each sample were separated via SDS-PAGE on 12% polyacrylamide gels and subsequently transferred to polyvinylidene difluoride (PVDF) membranes using a wet transfer system [45,46]. Membranes were blocked with 5% non-fat dry milk in Tris-buffered saline containing 0.1% Tween-20 (TBST) for 1 h at room temperature to reduce non-specific binding [45].

The membranes were incubated overnight at 4 °C with primary antibodies targeting Bcl-2, Pro-caspase-3, Beclin-1, LC3 (LC3A/B), and GAPDH (1:1000 dilution; Cell Signaling Technology, Danvers, MA, USA). After washing, membranes were incubated with appropriate horseradish peroxidase-conjugated secondary antibodies (1:2000) for 1 h at room temperature. Signal detection was performed using enhanced chemiluminescence (ECL) substrate (Thermo Fisher), and band intensities were visualized using a ChemiDoc imaging system (Bio-Rad, Hercules, CA, USA).

Western blotting was used to evaluate protein expression of Bcl-2, Pro-caspase-3, Beclin-1, and LC3-I/II [47]. Band intensities were analyzed using ImageJ software and normalized to GAPDH. Western blot analyses were conducted using *n* = 3 independent biological replicates, each obtained from separate chondrocyte culture passages. Densitometric quantification therefore reflects biological variability rather than technical repetition.

### 4.10. Statistical Analysis

All quantitative data were expressed as mean ± standard deviation (SD). Western blot analyses were conducted using *n* = 3 independent biological replicates, and densitometric quantification of protein bands was performed in triplicate for each replicate to ensure analytical consistency. For fluorescence-based assays (Rhodamine 123, Acridine Orange, and Propidium Iodide staining), each treatment condition was analyzed across *n* = 3 independent biological replicates, with fluorescence intensity measurements acquired in triplicate within each replicate. 

Statistical analyses were carried out using GraphPad Prism version 8.4.2 (GraphPad Software, San Diego, CA, USA). Comparisons among multiple groups were performed using one-way analysis of variance (ANOVA) followed by Tukey’s post hoc test for pairwise analysis. A *p*-value < 0.05 was considered statistically significant. Graphs were generated using the same software. This analytical approach ensured reproducibility, minimized intra-sample variation, and strengthened the statistical reliability of the data.

## 5. Conclusions

In this study, curcumin mitigated LPS-induced cellular stress in osteoarthritic chondrocytes by maintaining mitochondrial homeostasis, reducing caspase activation, and promoting autophagic flux. These effects were supported by fluorescence imaging and Western blot analyses of key apoptotic (pro-caspase-3, Bcl-2) and autophagic (Beclin-1, LC3-II) markers. Curcumin demonstrated a balanced regulatory effect, with the 25 µM concentration eliciting strong cytoprotective and anti-apoptotic responses, while the 100 µM concentration sustained this protection through feedback regulation between apoptosis and autophagy. Collectively, the findings indicate that curcumin exerts an integrated, rather than strictly dose-dependent, modulation of cell survival pathways. This dual anti-apoptotic and pro-autophagic role underscores its promise as a multi-targeted therapeutic candidate for osteoarthritis. Further in vivo studies and clinical trials are warranted to validate these in vitro results and explore curcumin’s translational potential in OA treatment.

## Data Availability

The raw data (including image analysis spreadsheets and raw Western blot images with molecular weight markers) supporting the conclusions of this article will be made available by the corresponding author on request.

## References

[1] Zeng N., Yan Z.-P., Chen X.-Y., Ni G.-X. (2020). Infrapatellar Fat Pad and Knee Osteoarthritis. Aging Dis..

[2] Neogi T. (2013). The Epidemiology and Impact of Pain in Osteoarthritis. Osteoarthr. Cartil. OARS Osteoarthr. Res. Soc..

[3] Pettenuzzo S., Berardo A., Belluzzi E., Pozzuoli A., Ruggieri P., Carniel E.L., Fontanella C.G. (2025). Mechanical insights into fat pads: A comparative study of infrapatellar and suprapatellar fat pads in osteoarthritis. Connect. Tissue Res..

[4] Zhao Z., Li Y., Wang M., Zhao S., Zhao Z., Fang J. (2020). Mechanotransduction pathways in the regulation of cartilage chondrocyte homoeostasis. J. Cell. Mol. Med..

[5] Rahman M.M., Watton P.N., Neu C.P., Pierce D.M. (2023). A chemo-mechano-biological modeling framework for cartilage evolving in health, disease, injury, and treatment. Comput. Methods Programs Biomed..

[6] Fellows C.R., Matta C., Zakany R., Khan I.M., Mobasheri A. (2016). Adipose, Bone Marrow and Synovial Joint-Derived Mesenchymal Stem Cells for Cartilage Repair. Front. Genet..

[7] Li G., Yin J., Gao J., Cheng T.S., Pavlos N.J., Zhang C., Zheng M.H. (2013). Subchondral bone in osteoarthritis: Insight into risk factors and microstructural changes. Arthritis Res. Ther..

[8] Palazzo C., Nguyen C., Lefevre-Colau M.-M., Rannou F., Poiraudeau S. (2016). Risk factors and burden of osteoarthritis. Ann. Phys. Rehabil. Med..

[9] Li H., Yuan S., Yue Z., Zhang L., Chen S., Qian Q., Fu Q., Chen Y. (2025). Suppressive effect of curcumin on apoptosis of articular chondrocytes via regulation on NF-κB pathway and NLRP3 inflammasome. Cytotechnology.

[10] Zhang M., Wu J., Cai K., Liu Y., Lu B., Zhang J., Xu J., Gu C., Chen T. (2024). From dysfunction to healing: Advances in mitochondrial therapy for Osteoarthritis. J. Transl. Med..

[11] Jiang E., Chen X., Bi Y., Pan C., Li X., Lan X. (2024). Curcumin Inhibits Oxidative Stress and Apoptosis Induced by H2O2 in Bovine Adipose-Derived Stem Cells (bADSCs). Animals.

[12] He Y., Li Z., Alexander P.G., Ocasio-Nieves B.D., Yocum L., Lin H., Tuan R.S. (2020). Pathogenesis of Osteoarthritis: Risk Factors, Regulatory Pathways in Chondrocytes, and Experimental Models. Biology.

[13] Guo P., Alhaskawi A., Adel Abdo Moqbel S., Pan Z. (2025). Recent development of mitochondrial metabolism and dysfunction in osteoarthritis. Front. Pharmacol..

[14] Polley S. (2025). Illuminating Cancer Metabolism: Unveiling Mitochondrial Secrets Through Fluorescence Microscopy. FocalPlane. https://focalplane.biologists.com/2025/03/23/illuminating-cancer-metabolism-unveiling-mitochondrial-secrets-through-fluorescence-microscopy/.

[15] Cao H., Zhou X., Xu B., Hu H., Guo J., Wang M., Li N., Jun Z. (2024). Advances in the study of mitophagy in osteoarthritis. J. Zhejiang Univ. Sci. B.

[16] Sathyabhama M., Priya Dharshini L.C., Karthikeyan A., Kalaiselvi S., Min T. (2022). The Credible Role of Curcumin in Oxidative Stress-Mediated Mitochondrial Dysfunction in Mammals. Biomolecules.

[17] Rahn C.A., Bombick D.W., Doolittle D.J. (1991). Assessment of mitochondrial membrane potential as an indicator of cytotoxicity. Fundam. Appl. Toxicol. Off. J. Soc. Toxicol..

[18] Hou D., Liao H., Hao S., Liu R., Huang H., Duan C. (2024). Curcumin simultaneously improves mitochondrial dynamics and myocardial cell bioenergy after sepsis via the SIRT1-DRP1/PGC-1α pathway. Heliyon.

[19] Cho K.B., Park C.H., Kim J., Tin T.D., Kwak S.-H. (2020). Protective role of curcumin against lipopolysaccharide-induced inflammation and apoptosis in human neutrophil. Anesth. Pain Med..

[20] Paglin S., Hollister T., Delohery T., Hackett N., McMahill M., Sphicas E., Domingo D., Yahalom J. (2001). A novel response of cancer cells to radiation involves autophagy and formation of acidic vesicles. Cancer Res..

[21] Sala de Oyanguren F.J., Rainey N.E., Moustapha A., Saric A., Sureau F., O’Connor J.-E., Petit P.X. (2020). Highlighting Curcumin-Induced Crosstalk between Autophagy and Apoptosis as Supported by Its Specific Subcellular Localization. Cells.

[22] Bao Q., Wang Z., Yang T., Su X., Chen Y., Liu L., Deng Q., Liu Q., Shao C., Zhu W. (2025). Curcumin induces mitochondrial dysfunction-associated oxidative DNA damage in ovarian cancer cells. PLoS ONE.

[23] Lu G., Wang Y., Shi Y., Zhang Z., Huang C., He W., Wang C., Shen H. (2022). Autophagy in health and disease: From molecular mechanisms to therapeutic target. MedComm.

[24] Boșca A.B., Ilea A., Sorițău O., Tatomir C., Miklášová N., Pârvu A.E., Mihu C.M., Melincovici C.S., Fischer-Fodor E. (2019). Modulatory effect of curcumin analogs on the activation of metalloproteinases in human periodontal stem cells. Eur. J. Oral Sci..

[25] Crowley L.C., Marfell B.J., Scott A.P., Boughaba J.A., Chojnowski G., Christensen M.E., Waterhouse N.J. (2016). Dead Cert: Measuring Cell Death. Cold Spring Harb. Protoc..

[26] Gupta R., Ambasta R.K. (2021). Pravir Kumar Autophagy and apoptosis cascade: Which is more prominent in neuronal death?. Cell. Mol. Life Sci..

[27] Zhang P., Fang J., Zhang J., Ding S., Gan D. (2020). Curcumin Inhibited Podocyte Cell Apoptosis and Accelerated Cell Autophagy in Diabetic Nephropathy via Regulating Beclin1/UVRAG/Bcl2. Diabetes Metab. Syndr. Obes. Targets Ther..

[28] Patnaik R., Jannati S., Sivani B.M., Rizzo M., Naidoo N., Banerjee Y. (2023). Efficient Generation of Chondrocytes From Bone Marrow-Derived Mesenchymal Stem Cells in a 3D Culture System: Protocol for a Practical Model for Assessing Anti-Inflammatory Therapies. JMIR Res. Protoc..

[29] Alcian Blue—An Overview|ScienceDirect Topics. https://www.sciencedirect.com/topics/medicine-and-dentistry/alcian-blue.

[30] Sridharan G., Shankar A.A. (2012). Toluidine blue: A review of its chemistry and clinical utility. J. Oral Maxillofac. Pathol..

[31] 26904 RhoAGTP-IHC and IF 02. NewEast Biosciences—GTPase Oncogene Bioact. Protein. https://neweastbio.com/26904-ihc-02/.

[32] Wang G., An Y., Zhang X., Ding P., Bi H., Zhao Z. (2021). Chondrocyte Spheroids Laden in GelMA/HAMA Hybrid Hydrogel for Tissue-Engineered Cartilage with Enhanced Proliferation, Better Phenotype Maintenance, and Natural Morphological Structure. Gels.

[33] Suzuki Y., Hasegawa H., Tsuji T., Tsuruda K., Sasaki D., Ishihara K., Nagai K., Yanagihara K., Yamada Y., Kamihira S. (2013). Relationships of diverse apoptotic death process patterns to mitochondrial membrane potential (ΔΨm) evaluated by three-parameter flow cytometric analysis. Cytotechnology.

[34] Rovini A., Heslop K., Hunt E.G., Morris M.E., Fang D., Gooz M., Gerencser A.A., Maldonado E.N. (2021). Quantitative analysis of mitochondrial membrane potential heterogeneity in unsynchronized and synchronized cancer cells. FASEB J. Off. Publ. Fed. Am. Soc. Exp. Biol..

[35] Baracca A., Sgarbi G., Solaini G., Lenaz G. (2003). Rhodamine 123 as a probe of mitochondrial membrane potential: Evaluation of proton flux through F_0_ during ATP synthesis. Biochim. Biophys. Acta.

[36] D’Ascola A., Irrera N., Ettari R., Bitto A., Pallio G., Mannino F., Atteritano M., Campo G.M., Minutoli L., Arcoraci V. (2019). Exploiting Curcumin Synergy With Natural Products Using Quantitative Analysis of Dose–Effect Relationships in an Experimental In Vitro Model of Osteoarthritis. Front. Pharmacol..

[37] Patnaik R., Varghese R., Jannati S., Naidoo N., Banerjee Y. (2024). Targeting PAR2-mediated inflammation in osteoarthritis: A comprehensive in vitro evaluation of oleocanthal’s potential as a functional food intervention for chondrocyte protection and anti-inflammatory effects. BMC Musculoskelet. Disord..

[38] Thomé M.P., Filippi-Chiela E.C., Villodre E.S., Migliavaca C.B., Onzi G.R., Felipe K.B., Lenz G. (2016). Ratiometric analysis of Acridine Orange staining in the study of acidic organelles and autophagy. J. Cell Sci..

[39] Wang X., Xiong T., Cui M., Li N., Li Q., Zhu L., Duan S., Wang Y., Guo Y. (2021). A novel targeted co-delivery nanosystem for enhanced ovarian cancer treatment via multidrug resistance reversion and mTOR-mediated signaling pathway. J. Nanobiotechnol..

[40] Crowley L.C., Scott A.P., Marfell B.J., Boughaba J.A., Chojnowski G., Waterhouse N.J. (2016). Measuring Cell Death by Propidium Iodide Uptake and Flow Cytometry. Cold Spring Harb. Protoc..

[41] Kim L., Kim J.Y. (2018). Chondroprotective effect of curcumin and lecithin complex in human chondrocytes stimulated by IL-1β via an anti-inflammatory mechanism. Food Sci. Biotechnol..

[42] Hussaini S.M.Q., Jun H., Cho C.H., Kim H.J., Kim W.R., Jang M.-H. (2013). Heat-Induced Antigen Retrieval: An Effective Method to Detect and Identify Progenitor Cell Types during Adult Hippocampal Neurogenesis. J. Vis. Exp..

[43] Li X., Feng K., Li J., Yu D., Fan Q., Tang T., Yao X., Wang X. (2017). Curcumin Inhibits Apoptosis of Chondrocytes through Activation ERK1/2 Signaling Pathways Induced Autophagy. Nutrients.

[44] Jiang C., Luo P., Li X., Liu P., Li Y., Xu J. (2020). Nrf2/ARE is a key pathway for curcumin-mediated protection of TMJ chondrocytes from oxidative stress and inflammation. Cell Stress Chaperones.

[45] Mahmood T., Yang P.-C. (2012). Western blot: Technique, theory, and trouble shooting. N. Am. J. Med. Sci..

[46] Towbin H., Staehelin T., Gordon J. (1979). Electrophoretic transfer of proteins from polyacrylamide gels to nitrocellulose sheets: Procedure and some applications. Proc. Natl. Acad. Sci. USA.

[47] Shakibaei M., Schulze-Tanzil G., John T., Mobasheri A. (2005). Curcumin protects human chondrocytes from IL-l1beta-induced inhibition of collagen type II and beta1-integrin expression and activation of caspase-3: An immunomorphological study. Ann. Anat. Anat. Anz. Off. Organ Anat. Ges..

